# Lifestyles, arterial aging, and its relationship with the intestinal and oral microbiota (MIVAS III study): a research protocol for a cross-sectional multicenter study

**DOI:** 10.3389/fpubh.2023.1164453

**Published:** 2023-06-29

**Authors:** Cristina Lugones-Sánchez, Sandra Santos-Mínguez, Rita Salvado, Susana González-Sánchez, Olaya Tamayo-Morales, Amaya Hoya-González, José I. Ramírez-Manent, Rosa Magallón-Botaya, José A. Quesada-Rico, Miriam D. Garcia-Cubillas, Emiliano Rodríguez-Sánchez, Manuel A. Gómez-Marcos, Rocío Benito-Sanchez, Alex Mira, Jesus M. Hernandez-Rivas, Luis Garcia-Ortiz, MIVAS III Researchers Group

**Affiliations:** ^1^Primary Care Research Unit of Salamanca (APISAL), Salamanca Primary Healthcare Management, Castilla y León Regional Health Authority (SACyL), Institute of Biomedical Research of Salamanca (IBSAL), Salamanca, Spain; ^2^Research Network on Chronicity, Primary Care and Health Promotion (RICAPPS), Salamanca, Spain; ^3^Cancer Research Centre, Institute of Biomedical Research of Salamanca (IBSAL), Institute of Molecular and Cellular Biology of Cancer (IBMCC), University of Salamanca-CSIC, Salamanca, Spain; ^4^Calvià Primary Care Center, Balearic Islands Health Research Institute (IDIBSA), Health Service of Balearic Islands, Calvià, Spain; ^5^Department of Medicine, University of the Balearic Islands, Palma de Mallorca, Spain; ^6^Institute for Health Research Aragón (IIS Aragón), Zaragoza, Spain; ^7^Department of Medicine, Psychiatry and Dermatology, University of Zaragoza, Zaragoza, Spain; ^8^Department of Clinical Medicine, Miguel Hernandez University of Elche, Sant Joan d'Alacant, Spain; ^9^Department of Medicine, University of Salamanca, Salamanca, Spain; ^10^Department of Health and Genomics, FISABIO Foundation, Valencia, Spain; ^11^CIBER Center for Epidemiology and Public Health, Madrid, Spain; ^12^Haematology Department, Institute of Biomedical Research of Salamanca (IBSAL), University Hospital of Salamanca, Salamanca, Spain; ^13^Department of Biomedical and Diagnostic Sciences, University of Salamanca, Salamanca, Spain

**Keywords:** exercise, diet, smoking, gastrointestinal microbiome, oral microbiome, vascular stiffness, atherosclerosis, cognitive dysfunction

## Abstract

**Background:**

The microbiota is increasingly recognized as a significant factor in the pathophysiology of many diseases, including cardiometabolic diseases, with lifestyles probably exerting the greatest influence on the composition of the human microbiome. The main objectives of the study are to analyze the association of lifestyles (diet, physical activity, tobacco, and alcohol) with the gut and oral microbiota, arterial aging, and cognitive function in subjects without cardiovascular disease in the Iberian Peninsula. In addition, the study will examine the mediating role of the microbiome in mediating the association between lifestyles and arterial aging as well as cognitive function.

**Methods and analysis:**

MIVAS III is a multicenter cross-sectional study that will take place in the Iberian Peninsula. One thousand subjects aged between 45 and 74 years without cardiovascular disease will be selected. The main variables are demographic information, anthropometric measurements, and habits (tobacco and alcohol). Dietary patterns will be assessed using a frequency consumption questionnaire (FFQ) and the Mediterranean diet adherence questionnaire. Physical activity levels will be evaluated using the International Physical Activity Questionnaire (IPAQ), Marshall Questionnaire, and an Accelerometer (Actigraph). Body composition will be measured using the Inbody 230 impedance meter. Arterial aging will be assessed through various means, including measuring medium intimate carotid thickness using the Sonosite Micromax, conducting analysis with pulse wave velocity (PWA), and measuring pulse wave velocity (cf-PWV) using the Sphygmocor System. Additional cardiovascular indicators such as Cardio Ankle Vascular Index (CAVI), ba-PWV, and ankle-brachial index (Vasera VS-2000^®^) will also be examined. The study will analyze the intestinal microbiota using the OMNIgene GUT kit (OMR−200) and profile the microbiome through massive sequencing of the 16S rRNA gene. Linear discriminant analysis (LDA), effect size (LEfSe), and compositional analysis, such as ANCOM-BC, will be used to identify differentially abundant taxa between groups. After rarefying the samples, further analyses will be conducted using MicrobiomeAnalyst and R v.4.2.1 software. These analyses will include various aspects, such as assessing α and β diversity, conducting abundance profiling, and performing clustering analysis.

**Discussion:**

Lifestyle acts as a modifier of microbiota composition. However, there are no conclusive results demonstrating the mediating effect of the microbiota in the relationship between lifestyles and cardiovascular diseases. Understanding this relationship may facilitate the implementation of strategies for improving population health by modifying the gut and oral microbiota.

**Trial registration:**

clinicaltrials.gov/ct2/show/NCT04924907, ClinicalTrials.gov, identifier: NCT04924907. Registered on 21 April 2021.

## 1. Introduction

The gut microbiota consists of a diverse community of 10^13^ to 10^14^ bacteria, archaea, and eukaryotes, with the number of genes represented being more than two orders of magnitude greater than the human genome (1). Although relatively stable in adults, the gut microbiome can be altered by diet and medications (2). Most of the microbial populations belong to the domain of bacteria, with ~90% belonging to the phyla *Bacteroidetes* and *Firmicutes* (3). Metabolites produced by gut bacteria include short-chain fatty acids, which can provide ~10% of the daily energy requirement in humans, regulate glucose homeostasis and cholesterol metabolism, and modulate the immune system (4). Bile acids, which are involved in the absorption of fat-soluble vitamins, regulate triglycerides and contribute to the maintenance of intestinal barrier function, in addition to exerting antimicrobial effects depending on their type and concentration (5). Lipopolysaccharides are associated with insulin resistance and impaired intestinal homeostasis, leading to increased permeability of the intestinal membrane that allows bacteria to translocate, activating the immune system and inducing inflammation (6). The oral cavity is the gateway to the digestive and respiratory systems and is highly vascularized, indicating the possible involvement of the oral microbiome in some systemic diseases such as cardiovascular, endocrine, cancer, and other diseases; the most prevalent phyla are *Firmicutes, Proteobacteria, Actinobacteria, Bacteroides, Fusobacteria*, and *Spirochetes* (7).

Although intestinal architecture, genetically defined traits, and age are the factors most strongly influencing the composition of the intestinal microbiome (8), diet and lifestyle are likely the major causes of interindividual variation in its composition in humans. A multitude of factors, such as restrictive diets and strict vegan or gluten-free diets, have been shown to change the intestinal microbiota and can contribute to intestinal dysbiosis (3). Obesity is related to reduced fecal microbial diversity, just as a high-salt diet leads to dysbiosis (3). Physical exercise also has a positive impact on the biodiversity of the microbiota, as some studies on animals and humans have shown (4, 9). Nevertheless, while evidence suggests that the microbiota plays a key role in maintaining systemic nitric oxide (NO) homeostasis, causal inferences regarding healthy aging are limited (10). There are also no conclusive studies analyzing the relationship between oral microbiomes and different lifestyles.

The gut microbiome changes across the lifespan, reaching maturity at 2–3 years, remaining relatively stable during adulthood, and beginning to change as the host starts to age (11). During the aging process, the gut microbiome becomes unstable and experiences a decrease in diversity (12). The loss of bacterial diversity in the intestine of older adults has been associated with increased frailty and reduced cognitive performance, as well as the institutionalization of aging individuals and reduced physical activity (13). Dysbiosis has been associated with several diseases and conditions prevalent in older adults, including type 2 diabetes, frailty, insulin resistance, atherosclerosis, hypertension, Alzheimer's disease, and others (4). Some associations have also been found with parameters of arterial aging, such as an increase in intima-media thickness (IMT) with the abundance of *Serratia* and *Blautia* and a greater presence of *Bacteriodes* associated with a higher pulse wave velocity in subjects without diabetes (14). In a cohort of women in the UK, an inverse association between the diversity of the intestinal microbiota and arterial stiffness was found (15). Although there are no specific studies on the oral microbiome and vascular aging, evidence would suggest that oral bacteria play an important role in mediating the beneficial effects of nitrate-rich foods on blood pressure (16) and, thus, vascular function.

Dysbiosis is characterized by the presence of more proinflammatory species that favor the development of metabolic diseases, either by independent or diet-dependent mechanisms. It can affect the metabolic state of the organism in addition to significantly impacting blood pressure, glycemia, and atherosclerosis, which are all cardiovascular risk factors (17). Such dysbiosis can increase intestinal permeability and favor the subsequent displacement of molecules produced by the intestinal microbiota, for example, phosphatidylcholine, choline, betaine, and L-carnitine, which are abundant in red meat and dairy products and are converted into trimethylamine, which is associated with the development and progression of cardiovascular disease (18). The composition of the intestinal microbiota is also linked to the early stages of hypertension (19), obesity, insulin resistance, metabolic syndrome, and type 2 diabetes (4). A meta-analysis (20) has confirmed the presence of 23 oral commensal bacteria, either individually or in coexistence, within atherosclerotic plaques in patients undergoing carotid endarterectomy, catheter atherectomy, or similar procedures. Nevertheless, the precise relationship between oral microbiota and atherosclerotic disease is yet to be fully clarified.

A mounting body of evidence suggests that altered gut microbiome composition is involved in the development of adipose tissue dysfunction and insulin resistance (21). In recent years, the intestinal microbiota has also been found to be involved in responses to type 2 diabetes mellitus treatment (22), with drug-induced metabolites transforming the structure of the gut microbiota (23). A recent epidemiological study indicated an association between mouthwash use and an elevated risk of developing prediabetes and diabetes, presumably by killing nitrate-reducing bacteria that can affect nitric oxide availability (24).

Microbial dysbiosis can lead to atherosclerosis, cerebrovascular disease, and endothelial dysfunction, which are risk factors for vascular cognitive impairment (18). In patients with Alzheimer's disease, the number of *Firmicutes* and *Actinobacteria* has been shown to decrease while those of *Bacteroidetes* increase, with a parallel decrease in *Eubacterium* and an increase in *Escherichia/Shigella*, defined as proinflammatory (25). However, the etiology of this fact remains unclear. Some longitudinal studies suggest a causal relationship between chronic periodontitis and the development of Alzheimer's disease (26). However, although a possible mediation of this relationship by the presence of oral gingipains in the brain has been posited, the explanation of how periodontitis or oral microbiome dysbiosis can lead to dementia remains unclear (26).

The study on position by the ESC Working Group on Coronary Pathophysiology and Microcirculation (27) points out that studies on the microbiome are hindered by the complexity of the measurements, as well as heterogeneity in terms of study design, methods used, sampling, parameters measured, and populations studied; furthermore, they are not sufficiently powerful as a rule to capture substantial variation in the gut microbiome. Moreover, to date, there is no universally accepted consensus as to what constitutes a healthy microbiome, and inter-individual variability is enormous. Thus, there remain many knowledge gaps regarding the factors influencing intestinal microbiota with a view to achieving a healthier profile. There is some evidence regarding the modification of lifestyles, but these results need to be confirmed by studies with greater power and population diversity. Meanwhile, although some studies hint at the relationship of the microbiota with arterial aging, the profile of a healthy cardiovascular and neurocognitive microbiota remains to be clearly established. Finally, little is known about the mediating role of the microbiota in the relationship of lifestyles with health, cardiology, and cerebrovascular disease.

For all these reasons, the objectives of this MIVAS III study are to analyze the relationship between lifestyles (eating patterns, regular physical activity, smoking, and drinking) and the gut and oral microbiota, along with anthropometric parameters and aging arterial, cardiovascular, and neurocognitive health in the general Spanish population. Similarly, the mediating role of the gut and oral microbiota in the relationship between lifestyles and vascular health will be analyzed. Gender differences in the association of lifestyles with the composition of the gut and oral microbiota and vascular structure and function will also be analyzed. In addition, the study will examine the opinions and experiences of the population through a discussion group to facilitate the transfer from the study to routine clinical practice.

## 2. Methods and analysis

### 2.1. Study design and setting

In its first phase, this study is designed as a multicenter cross-sectional study that aims to analyze associations between lifestyles and gut microbiota components, as well as the relationship between the gut microbiota and arterial aging. The second phase involves cohort follow-up. Here, discussion groups will bring out the opinions and experiences of the population regarding the influence of microbiota composition on health. The MIVAS III study (PI20/00321) was registered with ClinicalTrials.gov (registration number: NCT04924907) on 21 April 2021. This project is to be conducted within 3 years of Clinicaltrials.gov registration. The first 2 years will be spent conducting sample selection and data gathering through the questionnaires and explorations described below. In the third year, the analysis and dissemination of the results will be carried out. The study was approved by the “Committee of Ethics of Research with Medicines of the Health Area of Salamanca” on 13 November 2020 (cod. 2020 10 568). A SPIRIT (Standard Protocol Items: Recommendations for Interventional Trials) checklist (28) is available for this protocol (Supplementary material 1).

The study will be carried out in primary healthcare facilities within the scope of the Network for Research on Chronicity, Primary Care, Prevention, and Health Promotion (RICAPPS) in Spanish centers (Salamanca, Valladolid, Zaragoza, and Palma de Mallorca) and the Iberian network on arterial structure, central hemodynamics, and neurocognition, with the participation of Spanish and Portuguese researchers. Microbiota analysis will be carried out at the Cancer Research Center of Salamanca and in the Department of Health and Genomics of the University of Valencia.

### 2.2. Study population

One thousand participants will be selected from users of Spanish primary healthcare centers who meet the following inclusion criteria: being aged between 45 and 74 years and not meeting any of the exclusion criteria, which include the following: cardiovascular disease; body mass index (BMI) > 40 kg/m^2^; severe renal failure; chronic inflammatory or bowel disease; oncology disease under treatment; terminal state; pregnancy; and use of antibiotics in the previous 2 weeks. Participants will be recruited at health centers with the active participation of family doctors and nurses.

The sample size for this study was calculated with the free software GRANMO (http://www.imim.cat/ofertadeserveis/software-public/granmo/). The size necessary to detect the difference of 0.1 points in the alpha diversity of the microbiota (Shannon index) between the subjects that do or do not meet the Mediterranean diet criteria has been estimated. The proportion of compliance with the Mediterranean diet in the Evident II study was 30% (29). Therefore, assuming an alpha risk of 0.05 and a beta risk of 0.2 in a two-sided test, a common standard deviation of 0.4 (30), and a 5% dropout rate due to technical difficulties or refusal to participate, 190 subjects in the first group and 436 in the second are required to detect a difference ≥0.1 point between them in the Shannon index. On the other hand, assuming a rate of exposure of 0.3% (dysbiosis), in the control group, 471 subjects with vascular aging and 471 controls are required to detect a minimum odds ratio of 1.5 in the study factor (alpha diversity). Therefore, the 1,000 participants to be included in the study will be enough to test the mentioned hypotheses and will have an equal gender distribution.

While patients did not participate in the design of the study, they will actively participate in recruitment by disseminating the study objectives and inclusion criteria through their organizations. At the end of the study, in addition to sending a detailed report with the results of each patient, a dissemination session will be organized for all patients included in the study. Through discussion groups, the opinions and experiences of the population regarding the influence of microbiota composition on health will be investigated. Similarly, their attitudes and possible resistance will be considered for the transfer to clinical practice.

### 2.3. Variables

All assessments will be carried out within 7 days and will be monitored and quality controlled by a researcher independent of the sponsor.

#### 2.3.1. Sociodemographic variables

Data on age, sex, marital status, educational level, and occupation will be collected when participants are accepted into the study. A history of hypertension, dyslipidemia, diabetes, hypothyroidism, other diseases, and drug use will be documented.

#### 2.3.2. Anthropometric measurements

Body weight will be measured twice using an approved electronic scale (Seca 770 medical scale and measurement systems, Birmingham, United Kingdom), with the patient dressed in light clothing and barefoot. BMI will be calculated as the weight (kg) divided by the height squared (m^2^). The waist circumference will be measured with a flexible, graduated tape measure with the patient standing up and undressed. Body composition will be determined using the InBody 230 monitor (InBody Co. Ltd., Seoul, Korea), which provides body composition analysis information. Clinical blood pressure (BP) will be measured three times, with the average of the last two times being recorded, using a validated Omron M10-IT model sphygmomanometer (Omron Healthcare, Kyoto, Japan). Measurements will be made on the dominant arm of the participant in a sitting position after at least 5 min of rest with an appropriately sized cuff, which was determined by measuring the circumference of the upper arm and following the recommendations of the European Society of Hypertension (ESH) (31).

#### 2.3.3. Habits and lifestyles

##### 2.3.3.1. Diet

Participants' eating habits will be assessed using a 137-item semi-quantitative food frequency questionnaire (FFQ) previously validated in Spain (32). The FFQ is based on typical portion sizes that will be multiplied by the frequency of consumption of each food. The FFQ estimates food consumption frequency for the year before the interview and is divided into *nine* consumption frequency categories ranging from never to more than *six* servings per day. This will be used to estimate the daily intake of macronutrients and micronutrients.

The Mediterranean Diet Adherence Screener (MEDAS) (33), developed by the PREDIMED study group, will be used to assess adherence to the Mediterranean diet. Each question will be scored as 0 or 1. Adequate adherence to the Mediterranean diet will be assumed when the total score is ≥9 points. Food consumption will be recorded during a normal week with the application developed in the EVIDENT study (34) (registry number 00/2014/2207).

##### 2.3.3.2. Physical activity and sedentary behavior

Physical activity will be measured using both objective and subjective methods. *The ActiGraph-GT3X accelerometer* (ActiGraph, Shalimar, FL), which has been previously validated (35), will be used to measure the physical activity of the subjects for seven consecutive days, including step counts and levels of moderate-to-vigorous physical activity. The original data from the accelerometers will be collected at a frequency of 30 Hz. Before the test, the accelerometer will be initialized, and the correct way of wearing the accelerometer and matters needing attention will be discussed.

The specific requirements for wearing it are as follows: (a) the accelerometers are fixed at the waist and positioned on another axillary line at the iliac crest level of the right or left hip; (b) wearing the accelerometer continuously for 7 consecutive days except during bathing or swimming. The accelerometers will record activity during the day and at night. If the number of days is <3 days a week or the time of wearing is <8 h a day, the data will be considered invalid. Data will be recorded at 1-min intervals. The intensity of the physical activity (low, moderate, or high) will be determined according to the cut-off points proposed by Freedson et al. (36).

The *International Physical Activity Questionnaire-Short Form (IPAQ-SF*) (37) will also be used to assess physical activity. The short form (nine items) categorizes physical activity for the last 7 days at three levels of intensity: (1) intense physical activity, (2) moderate-intensity activity, and (3) light activity. The IPAQ is a self-reported questionnaire that evaluates sitting and active time in the last 7 days, differentiating between walking, moderate-intensity, and vigorous-intensity activities according to the energy expenditure estimated for each of them [3.3, 4.0, and 8.0 metabolic equivalents of task (MET), respectively].

Participants reported their sedentary behaviors in total daily sitting time using the *Marshall Sitting Questionnaire (MSQ)* (38). This validated questionnaire assesses sitting time in hours and minutes on weekdays and weekend days across five domains: transportation, work, television watching, at-home computer use, and leisure not specified in other domains. In daily sitting hours, SB was calculated by summing the sitting time for each domain.

##### 2.3.3.3. Tobacco and alcohol consumption

A standardized questionnaire will be used to assess tobacco and alcohol consumption. Smoking status will be assessed through a questionnaire of *four* standard questions adapted from the WHO MONICA study (39). Study participants will be classified as current smokers or non-smokers (never or >1 year without smoking). To assess drinking, a structured questionnaire will be used to record the number of alcoholic beverages drunk in the previous week, the grams/week consumed will be estimated, and the patient will be classified as teetotal, low risk, intermediate risk, or high risk according to the criteria of the Spanish Ministry of Health (40).

#### 2.3.4. Vascular structure and function

##### 2.3.4.1. Carotid-femoral pulse wave velocity and central augmentation index

These parameters will be estimated using the SphygmoCor System (AtCor Medical Pty Ltd., Head Office, West Ryde, Australia). Carotid and femoral artery pulse waves will be analyzed with the patient in a supine position using the SphygmoCor System, estimating the delay compared with the ECG wave and calculating carotid-femoral pulse wave velocity (cf-PWV) in m/s. Distance will be measured with a measuring tape from the sternal notch to the carotid and femoral arteries at the sensor location. With the patient sitting and resting his/her arm on a rigid surface, pulse wave analysis will be performed with a sensor in the radial artery, using a mathematical transformation to estimate the aortic pulse wave and Central Augmentation Index (CAIx) (41).

##### 2.3.4.2. Cardio-ankle vascular index, brachial-ankle PWV, and ankle-brachial index

These parameters will be estimated using the VaSera device VS-2000 (Fukuda Denshi Co, Ltd., Tokyo, Japan). Cardio-ankle vascular index (CAVI) values will be automatically calculated by substituting the stiffness parameters in the following equation to detect the vascular elasticity and the cardio ankle PWV: stiffness parameter β = 2ρ × 1/(Ps–Pd) × ln (Ps/Pd) × PWV 2, where ρ is the blood density, Ps and Pd are SBP and DBP in mmHg, and PWV is measured between the aortic valve and ankle. The brachial-ankle PWV (ba-PWV) will be estimated using the following equation: ba-PWV = {[0.5934 × height (cm) + 14.4724]}/tba, where tba is the time the same waves were transmitted to the ankle (42). For this study, the mean ankle-brachial index (ABI), CAVI, and ba-PWV obtained will be considered. CAVI will be classified as normal (CAVI < 8), borderline (8 ≤ CAVI < 9), and abnormal (CAVI ≥ 9) (43).

##### 2.3.4.3. Central and peripheral augmentation index by the wrist-worn device

The wrist-worn device was developed by Microsoft Research (Redmond, Washington, USA) and validated by our team (44). We will use this device to make a short recording of the radial pulse wave, from which peripheral augmentation index (PAIx) and CAIx will be obtained. PAIx will be calculated as [second peak SBP (SBP2)–]/(first peak SBP-DBP) x 100 to yield a percentage (%) value, and CAIx will be calculated using the following formula: central augmentation pressure x 100/pulse pressure. Participants will be examined in a seated position, with the arm resting on a firm surface at heart level, after 10 min of rest.

##### 2.3.4.4. Assessment of vascular structure by carotid intima-media thickness

Measurements of carotid intima-media thickness (C- IMT) will be made by investigators trained in the common carotid after the examination of a 10 mm longitudinal section at 1 cm from the bifurcation. They will perform measurements in the proximal and distal walls of the lateral, anterior, and posterior projections, following an axis perpendicular to the artery to discriminate two lines, one for the intima blood interface and the other for the media-adventitious interface. The measurements will be obtained with the participant lying down, with the head extended and slightly turned opposite to the examined carotid artery. Following the ESC/ESH Guidelines for the Management of Arterial Hypertension (31), the measurement is considered pathological if the C-IMI <0.9 mm, the atheromatous plaque diameter is >1.5 mm, or the focal increase is 0.5 mm or 50% of the adjacent IMT (31).

#### 2.3.5. Target organ damage

##### 2.3.5.1. Renal assessment

Renal damage will be assessed by the estimated glomerular filtration rate using the Chronic Kidney Disease Epidemiology Collaboration (CKD-EPI) (45) and albumin–creatinine ratio, following the criteria of the ESC/ESH Guidelines for the Management of Arterial Hypertension (31).

##### 2.3.5.2. Cardiac assessment

Cardiac damage will be assessed using an ECG device. Left ventricular hypertrophy will be defined as a Sokolow-Lyon index > 3.5 mV or Cornell VDP > 2,440 mV × ms (31).

##### 2.3.5.3. Retinal vascular evaluation

Nasal and temporal images centered in the papilla, taken in a sitting position, will be obtained by a trained nurse using a non-mydriatic retinograph, TOPCON TRC NW 200 (Topcon Europe BC, Capelle aan den IJssel, The Netherlands). Using the ALTAIR software (registry entry 00/2015/995) specifically developed by our group, vessel thickness, area, and length of the retina were then calculated semiautomatically (46).

##### 2.3.5.4. Cognitive assessment

The Montreal Cognitive Assessment (MoCA), validated in Spain (47), will be used to evaluate cognitive impairment. The MoCA assesses several cognitive domains: attention and concentration, executive functions, memory, language, visuoconstructional skills, conceptual thinking, calculations, and orientation. The total possible score will be 30 points; a score of 26 or above will be considered normal. The time estimated for MoCA administration will be approximately 10 min.

#### 2.3.6. Laboratory measurements

Venous blood samples will be collected between 08:00 and 09:00 after fasting for 12 h. A complete blood count, fasting plasma glucose, creatinine, uric acid, liver function, lipids, inflammatory markers, and thyroid function will be measured using standard automated enzymatic methods. A blood sample from each participant will be frozen for subsequent evaluation of total bile acids, deoxycholic acid, and SCFA concentration (mg/mL).

#### 2.3.7. Gut and oral microbiota measurements

Participants will collect stool samples using the OMNIgene GUT (OMR-200) kit, which allows transport and storage while ensuring that stabilized DNA is preserved so that the *in vivo* microbiota profile will be accurately represented. The OMNIgene GUT kit is an easy self-collection system, enabling participants to properly collect their own samples at home following the manufacturer's recommendations. This kit minimizes bias introduced by microbial growth and DNA degradation, obtaining high-quality DNA appropriate for 16S rRNA gene microbiome profiling. During the study visit, unstimulated saliva samples will also be collected by spitting into a sterile 50 ml Falcon tube (*GenoChem World*). Subsequently, 2 ml of this sample will be transferred with a sterile pipette to two different and clearly labeled microtubes (*criogenic vial*), with 1 ml in each microtube. Once stool and saliva samples have been collected, they will be frozen at−20°C and sent to the Cancer Research Institute, maintaining the storage conditions.

According to the manufacturer's instructions, DNA will be extracted from a total volume of 100–150 ul of each feces sample using the FastDNA Soil kit [FastDNA^®^ SPIN Kit for Soil (MP Biomedicals, USA)]. Each sample volume will be added together with MT buffer and sodium phosphate buffer to a Lysing Matrix E tube (each tube contains 1.4 mm ceramic spheres, 0.1 mm silica spheres, and one 4 mm glass bead). This mixture will be homogenized for 40 s at 6 m/s in the FastPrep-24^TM^ 5G bead-beating grinder and lysis system, enabling a mechanical disruption of the living organisms' cell walls while protecting and solubilizing nucleic acids. DNA from saliva samples will be extracted with the MagNA Pure LC 2.0 Instrument (Roche Diagnostics, Barcelona, Spain) using the MagNA Pure LC DNA Isolation Kit III for Bacteria and Fungi (Roche Diagnostics GmbH) following the manufacturer's instructions with an additional enzymatic lysis step with lysozyme (20 mg/ml, 37°C, 60 min; Thermomixer comfort, Eppendorf), lysostaphin (2,000 units/mg protein, 37°C, 60 min; Sigma-Aldrich) and mutanolysin (4,000 units/mg protein, 37°C, 60 min; Sigma-Aldrich), following Dzidic et al. (48). DNA will be eluted with 50–150 ul of DNase/Pyrogen-Free Water (DES). Nanodrops will then be used to spectrophotometrically measure purified DNA yield (ng ul-1) and verify its quality and quantity. Low absorption ratios at 260/230 nm (<2) will be used as a marker for contamination from polysaccharides, and low absorption ratios at 260/280 nm (<1.7) will be used to identify protein impurities. Additionally, it will be fluorometrically quantified in a Qubit 4.0 fluorometer (Invitrogen, USA), and DNA integrity will be analyzed in a TapeStation 4,200 using Genomic DNA ScreenTapes (Agilent, USA). A high DNA integrity number (DIN) indicates large DNA fragments, whereas a low DIN indicates more fragmented DNA. The DIN scale ranges from 1 to 10.

##### 2.3.7.1. Amplicon library and Illumina sequencing of bacterial 16S rRNA genes

The 16S rRNA gene has been a mainstay of sequence-based bacterial taxonomic analysis for decades (49, 50). Variable regions v3-v4 have been shown to have an excellent resolution for phylogenetic classification, and their appropriate size for Illumina library preparation has made them the preferred region for high-throughput bacterial composition studies. However, some bacterial genera, such as *Streptococcus*, have been shown to be 100% identical in that region (48), and other bacterial genera, especially those with high G+C content, such as *Bifidobacterium*, have been shown to be under-amplified by the Illumina V3-V4 primers (51). In addition, variable regions v4-v6 have also been identified as the most functional regions in other studies (52). To select an appropriate region for our samples, we will perform a pilot study where 50 samples were amplified and sequenced using the V3-V4 and the V5-V6 regions. The corresponding taxonomic assignment showed high congruency between the two regions, with correlation coefficients of 0.957 to 0.979 at the genus level and 0.980 to 0.995 at the phylum level (*p* < 0.001 for all), indicating that both regions would provide similar bacterial composition assessments. However, 75% of the samples provided higher sequencing output using the V5-V6 region, with 25% of the samples reaching over 100,000 reads (vs. 4% when using the V3-V4 region). This would likely provide a complete assessment of bacterial richness and diversity, as low-frequency bacteria would only be detected with high sequencing coverage. On this basis, we selected the V5-V6 regions to be sequenced in this protocol.

Primers were taken from Gohl et al. (53) being the sequence: V5F_Nextera 5′-RGGATTAGATACCC-3′ and V6R_Nextera 5′-CGACRRCCATGCANCACCT-3′. These primer pairs have added Illumina adapter overhang nucleotide sequences:

ü Forward overhang: 5′ TCGTCGGCAGCGTCAGATGTGTATA AGAGACAG-[locus-specific sequence].
ü Reverse overhang: 5′ GTCTCGTGGGCTCGGAGATGTGTAT AAGAGACAG-[locus-specific sequence].

First, each sample will be amplified using these bacterial primers with Illumina adapters, as described above. Agencourt AMPure XP (Beckman Coulter) will be used to purify the resulting amplicons, which will then be amplified in a subsequent PCR where indexes are added. A different index sequence will be used to identify each sample from each patient. Finally, these indexed amplicons will be purified using the Agencourt AMPure XP kit. Amplicon libraries for each sample will then be generated. Equimolar amounts of these libraries should be pooled, normalized, and quantified in the Qubit. The pooled samples will be sequenced using an Illumina MiSeq platform (2 × 300 bp v3 chemistry).

### 2.4. Bioinformatics and statistical analysis

The data will be registered using the REDCap platform (Research Electronic Data Capture) (54, 55). The Kolmogorov-Smirnov test will be used to verify the normal distribution of the variables. Raw sequence data will be analyzed using an *in-house* pipeline. Quality passing-filter readings will be clustered into operational taxonomic units (OTUs). Quality control will be carried out on a *per-sample* basis, discarding paired ends with an overlap of <200 nt and removing chimeric sequences using *de novo* chimera detection in USEARCH (56). To analyze differences between qualitative variables, an X^2^ test will be used. To analyze the difference in means between quantitative variables in two categories, the Student's *t*-test or the Mann–Whitney U-test will be used, as appropriate. If the qualitative variable has more than two categories, an analysis of variance (ANOVA) will be used, and the results will be adjusted for the false discovery rate (FDR) using the Benjamin and Hochberg method. A *p*-value of 0.05 adjusted for FDR will be considered statistically significant. The Kruskal–Wallis test will be used if the variables are not normally distributed. An analysis of covariance (ANCOVA) will be performed to adjust for variables that may affect the results as confounding factors. The association of quantitative variables will be analyzed using Pearson's or Spearman's correlation test. We will fit linear or penalized multivariate logistic models using the Least Absolute Shrinkage and Selection Operator (LASSO) method (57), which can be applied to high-dimensional data to analyze the relationships between lifestyle variables and aging arterial, cardiovascular, and neurocognitive health status, as well as microbiota variables.

Given the compositional nature of microbiome data (58, 59), an ANCOM-BC method will also be used to identify significant differences in bacterial composition between groups and for frequency normalization. Linear discriminant analysis (LDA) effect size (LEfSe) will be used to identify differentially abundant taxa between groups using the Kruskal–Wallis test *P* < 0.05. These data will be validated using edgeR differential abundance analysis (false discovery rate adjusted *P* < 0.05 on species) on MicrobiomeAnalyst (60, 61). Further analyses will be performed using MicrobiomeAnalyst from the rarefied samples, including α and β diversity, abundance profiling, and clustering analysis. In the microbiota analysis, alpha, beta, and gamma diversity parameters will be estimated. Additionally, richness and equality parameters such as Chao1, Shannon, Simpson, and Pelou will be calculated. A description of all parameters will be developed, as will an exploratory analysis of the microbiota using graphs of abundance, richness, heatmaps, and phylogenetic graphs, as well as cluster analysis. Comparisons of diversity and taxa between groups will be made using the Welch test, Wilcoxon test, chi-square test (presence/absence), ANOVA procedure with Tukey's multiple comparisons, and Kruskal–Wallis test with FDR multiple comparisons, as appropriate. An exploratory compositional analysis of the microbiota will be carried out using biplot graphs, scree plots, dendrograms, and bar plots. The composition of the microbiota between groups will be compared using parametric procedures such as the *t*-test or ANOVA or non-parametric procedures, adjusting for multiple comparisons. To analyze the association of the microbiota with different groups with a multivariate approach, Zero-Hurdle Poisson Models (ZHP) will be adjusted given the predicted presence of excess zeros and overdispersion in the data. To analyze the associations between oral and intestinal microbiota and various groups, the Zero-Inflates Beta Regression Model (ZIBR) will be adjusted with random effects due to the predictable autocorrelation between the samples of the two microbiotas. To compare bacterial diversity between oral and gut samples, we will use Chao1 and Shannon index analyses using the same number of sequences per sample to control for biases in the estimations of richness and diversity, as described in Havsed et al. (62). To compare bacterial composition between oral and gut samples, paired samples will be used to study the correlations in bacterial levels intra- and inter-niche. To study the correlations among the bacteria, unsupervised sPCA from the mixOmics R package will be performed. A multivariate analysis (sPLS-canonical) from the mixOmics R package will also be applied using as input the normalized dataset of all bacteria counts, following Rohart et al. (63). Correlations will be conducted not only between bacteria but also between bacteria and different continuous variables, as described in Havsed et al. (62). Specifically, we will use sparse partial least squares (sPLS) to perform simultaneous variable selection in the two datasets (bacterial species abundance and quantitative variables). These associations will be plotted using heatmaps and networks with the mixOmics R package (63).

To examine whether the association between lifestyle and arterial aging is mediated by the intestinal microbiota, linear regression models will be fitted using bootstrapped mediation procedures included in the PROCESS SPSS macro (64). All variables will be analyzed disaggregated by sex, and differences with a gender perspective will be analyzed where appropriate since the influence of gender in many pathologies is known, particularly in cardio and cerebrovascular diseases.

The analyses will be conducted using the R v.4.2.1 program (65). R: A language and environment for statistical computing. R Foundation for Statistical Computing, Vienna, Austria (https://www.R-project.org/) and the statistical package SPSS for Windows version 28.0. (IBM, Armonk, New York: IBM Corp). A value of *p* < 0.05 will be considered statistically significant. Statisticians/researchers who perform different analyses will be blinded to patients' clinical data.

## 3. Discussion

This study aims to analyze the influence of different lifestyles on the composition of the gut and oral microbiomes. In addition, the influence of the microbiome on arterial aging and its impact on cardiovascular target organs, including the brain, will be evaluated by assessing neurocognition. Finally, the mediating role of the gut and oral microbiomes in the relationship between lifestyles, cardiovascular risk factors, and atherosclerotic disease will also be analyzed.

The human microbiota plays an essential role in health and the regulation of multiple physiological mechanisms (1). The concept of normal microbiota is still controversial since variability is important depending on multiple environmental and nutritional factors (30). In some studies, lifestyles have been shown to have a certain influence on the modification of the gut microbiota (66). Similarly, gut microbiota composition has also been implicated in the genesis of different diseases such as atherosclerosis (27), hypertension (67), obesity (68), insulin resistance, metabolic syndrome, and type 2 diabetes (69). In addition to the direct effects of diet on cardiovascular health, it is possible that it may exert effects through the gut microbiota, as has been observed in some cross-sectional and clinical trial studies (66), although the data are not conclusive. For example, a study with 893 participants estimated that the intestinal microbiota explained only 6% of the variance of triglycerides and 4% of HDL, regardless of age, sex, and genetic risk factors (70). On the other hand, good adherence to the Mediterranean diet has generally been associated with a beneficial gut microbiota composition (71).

The relationship between dietary patterns and the gut microbiome has been found in different studies, such as that of Latorre-Pérez et al. (30) in the general population, which confirms the association between some of the foods that characterize the Mediterranean diet and the abundance of bacterial taxa, and that of Wang et al. (72) in a diabetic population. Exercise has been linked to increased gut microbiota diversity in human cross-sectional studies (73). The beneficial effects of physical activity may be mediated, at least in part, by changes in the intestinal microbiota and its metabolites (74). However, in a study examining older men, the exercise program did not change composition compared to the baseline (75). Therefore, the relationship between exercise and microbiota is complex and insufficiently clear. Some studies have investigated the association of smoking with the gut microbiota, but they only showed a modest effect, and significant associations were not detected. Smoking may have indirect effects through alterations in the oral microbiota, and it is an important risk factor for periodontitis that is repeatedly associated with atherosclerotic vascular events (66). Low-grade inflammation has been proposed to be the cornerstone of different chronic diseases, such as metabolic syndrome (76), osteoarthritis (77), and type-2 diabetes (78). This inflammation has frequently been linked to an increase in intestinal permeability and a high intestinal translocation of proinflammatory mediators of bacterial origin, causing so-called “metabolic endotoxemia” and, therefore, the development of low-grade chronic inflammation and cardiovascular disease (79).

The MIVAS I (80) was a case-control study conducted in Spain (Salamanca) and Portugal (Guimaraes), with subjects from the EVA study (81) and the Cunha et al. (82), respectively, with the aim of analyzing the influences of gut microbiota on arterial stiffness. In this study, subjects with arterial stiffness and controls with the same characteristics were selected from the aforementioned studies. The MIVAS III study will select the general population of patients attended by family doctors and nurses in primary healthcare centers in different Spanish cities (Salamanca, Valladolid, Zaragoza, and Palma de Mallorca). The design is a multicenter cross-sectional study, with the main objective of analyzing the relationship between lifestyles (eating patterns, regular physical activity, smoking, and drinking) and the oral and gut microbiota. Second, the mediating role that the oral and gut microbiota may have in the relationship between lifestyle and arterial aging. In addition, the study will examine the opinions and experiences of the population through a discussion group to facilitate the transfer from the study to routine clinical practice. Finally, the second phase of the MIVAS III study will follow up on the cohort to analyze the effect of different oral and gut microbiota patterns on population health.

In conclusion, as previously discussed, healthy lifestyles, such as adequate dietary intake, a moderate or high level of physical activity, and not smoking, are beneficial for improving health and reducing the risk of many diseases, including cardiovascular diseases. It has also been found that lifestyles, particularly diet but also physical activity, act as modifiers of the composition and function of the microbiota. Nevertheless, no conclusive results have demonstrated the mediating effect of the microbiota in the relationship between lifestyles and cardiovascular diseases. Knowledge of the influence of different lifestyles on the composition of the gut and oral microbiome and of this on arterial aging and cardio- and cerebrovascular target organ lesions may facilitate the implementation of strategies to improve the health of the population by modifying the gut and oral microbiota.

The main limitations of the study are as follows: First, since it is not a random sample from the Iberian Peninsula, populational representation cannot be affirmed, although the fact that samples are collected in different locations across Spain could lend it a certain representativeness. Causality cannot be derived given the cross-sectional nature of the study, but it is possible to analyze the associations and generate hypotheses for future prospective etiological studies with cohort study designs and clinical trials. Nevertheless, the project has several strengths. With a sample of 1,000 participants from different parts of the Iberian Peninsula, it is larger than most of the published studies. Lifestyles and vascular health will be comprehensively assessed, in addition to the gut and oral microbiota. The participating clinical and basic researchers are from different areas of the Iberian Peninsula, which will facilitate the transfer of research results to the Spanish and Portuguese health systems as the first step in the internationalization of the project.

## 4. Summary

Evidence of the importance of microbiota in relation to multiple health processes is growing. Knowledge of which lifestyles can influence it can aid in the development of strategies to promote changes in diet, physical activity, and other habits that allow the microbiota to be modified to healthy profiles. This observational project is the first phase of future clinical trials that evaluate interventions on lifestyles to achieve healthy microbiota and, with it, reduce the risk of certain pathologies. It also addresses the relevant problem of how the different microbiome patterns are associated with vascular structure and function (arterial aging), damage to cardiovascular target organs, and cognitive impairment. Identifying microbiome patterns related to aging and highly prevalent processes will facilitate interventions to preventively modify them. Similarly, the mediating role of the microbiota in the relationship between lifestyle and arterial aging will be analyzed. Finally, the intention is also to contribute to the development of a methodology so that microbiota analysis can be transferred to clinical practice and contribute to the development of personalized medicine.

## Ethics statement

The studies involving human participants were reviewed and approved by the Committee of ethics of research with medicines of the health area of Salamanca on 13 November 2020 (cod. 2020 10 568). The patients/participants will be required to provide their written informed consent to participate in this study.

## Author contributions

LG-O and RS contributed substantially to the conception and design of the study. LG-O will have access to all the data in the study and will take responsibility for the integrity of the data, and the accuracy of data analysis and interpretation. CL-S and SS-M contributed to the drafting of the paper and LG-O and JH-R had the primary responsibility for the final approbation for the publication of the content. RS and JQ-R will contribute to the analysis and interpretation of quantitative data and OT-M will be responsible for discussion groups and qualitative analysis. MG-M, ER-S, AM, and LG-O have contributed to the critical review of the paper for important intellectual content. CL-S, AH-G, SG-S, RM-B, JR-M, and MG-C will be responsible for the collection and assembly of data. RM-B, SS-M, JH-R, and AM will be responsible for the genetic analysis of the intestinal and oral microbiomes. All authors have read and approved the final manuscript and they agree to be accountable for all aspects of the work, ensuring that questions related to the accuracy or integrity of any part of the work are appropriately investigated and resolved.
