# Supplementary material for: Lifestyles, arterial aging, and its relationship with the intestinal and oral microbiota (MIVAS III study): a research protocol for a cross-sectional multicenter study
Source: Front Public Health. 2023 Jun 29;11:1164453. doi: 10.3389/fpubh.2023.1164453 (PMC10344706; doi:10.3389/fpubh.2023.1164453)
Supplement: Supplementary file 2 [file Data_Sheet_2.PDF]

Supplementary Material

Lifestyles, arterial aging, and its relationship with the intestinal and oral microbiota (MIVAS III Study): A research protocol of a cross-sectional multi-center study

Running title: Lifestyles, arterial aging, and microbiota

Cristina Lugones-Sánchez [1,2†], Ana Garcia-Puente [1†], Rita Salvado[1], Susana González-Sánchez[1,2], Olaya Tamayo-Morales[1,2], José I Ramírez-Manet[2,3,4], Rosa Magallón-Botaya[2,5,6], José A Quesada-Rico[2,7], Miriam D Garcia Cubillas[1], Rocío Benito-Sánchez[8], Sandra Santos-Mínguez[8], Emiliano Rodríguez-Sánchez[1,2,9] Manuel A Gómez-Marcos[1,2,9], Alex Mira[10,11] Jesús M Hernández-Rivas [9,12] y Luis Garcia-Ortiz*[1,2,14] , and MIVAS III investigators group[2].

*** Correspondence:** Corresponding Author: Lgarciao@usal.es

INFORMATION SHEET AND INFORMED CONSENT

Lifestyles, arterial aging and its relationship with the intestinal microbiota (MIVAS III Study)

Introduction

We address you to inform you about a research study in which you are invited to participate. Your participation is voluntary. Please take the time you need to read the following information and consult what you want. Ask the researcher of this study if there is something that is not clear to you or if you want more information.

The purpose of the study

The main objectives of the study are to analyze the association of lifestyles (eating patterns, habitual physical activity, consumption of tobacco and alcohol) with the intestinal microbiota and this with arterial aging and cognitive function. In addition, the mediating role of the intestinal microbiota in the relationship of lifestyles with arterial aging and cognitive function in people without diagnosed cardiovascular diseases will be analyzed.

This research study has been approved by the Ethics Committee for Drug Research in Salamanca.

Study procedures and possible risks and discomforts

The researcher will assess whether you are a suitable candidate for this study. Once you have given your consent and the researcher has verified that you meet the criteria to participate in this study, you will be asked some questions about your health, physical activity and diet and the explorations that are detailed below will be performed: Weight determination , height, abdominal perimeter, central and peripheral blood pressure and ankle / arm index, study of vascular structure and function and retinography. The biological samples detailed below will also be obtained and will take an accelerometer for a week to assess physical activity. In general, only one visit of approximately one hour or more will be necessary to bring some samples that could not be collected on the first day.

Participation in this study would not cause any discomfort, and does not imply any risk to health, only the discomfort that may involve carrying out the tests, none of them invasive, the collection of urine and stool samples and blood collection. For most people, needle punctures for blood collection are no problem. However, although very rarely, they may cause minor bleeding, bruising, discomfort, infections and / or pain at the point of extraction.

Their participation in this study entails obtaining and using specific biological samples for research purposes, for which Law 14/2007 on biomedical research and Royal Decree 1716/2011 will be observed, regulations that guarantee respect for the rights granted to it. By signing this document, you agree that your samples be used for the purposes of this research study. A blood sample will be taken, in order to collect two samples (12 ml) that will be used specifically for the study, in the Primary Care Research Unit of Salamanca (APISAL), and a urine sample, which will be send to the University Hospital of Salamanca for processing. These will allow you to assess your lipid profile, glucose, creatinine, HBA1c and TSH and the albumin / creatinine index. A sample of stole and saliva will also be collected, which will be frozen in the Primary Care Research Unit of Salamanca and subsequently sent to the Institute of Molecular and Cellular Biology of Cancer Salamanca for the analysis of the intestinal microbiota by sequencing ribosomal RNA of the bacteria that compose it.

All information generated in this study will be stored, coded and used exclusively for the purposes specified here. The samples will be associated with a code that can only be related to you by the research team. Both your samples and your data will be kept under the appropriate security conditions and it is guaranteed that the subjects cannot be identified through means considered reasonable by people other than those authorized. Once the investigation is finished, the remaining samples will be destroyed, unless you consent so that they can be stored and used in future investigations. In case your data is transferred to other research groups, it will always be carried out according to current legislation, keeping your data coded, to carry out studies related to the objectives of this work, and with prior authorization of the Research Ethics Committee with Salamanca medicines.

Voluntary participation and withdrawal.You can freely decide whether or not to participate in this study, participation is completely voluntary. If you decide to participate, you still have the possibility of withdrawing at any time, without having to give explanations, and without any penalty or negative consequences for you. If you change your mind regarding your samples or your data, you have the right to request their destruction or anonymization, through the research team. However, you should know that the data obtained in the analyzes carried out up to that moment may be used for the requested purposes and may be kept in compliance with the corresponding legal obligations.

Possible benefits. The benefit for you will consist in knowing your peripheral and central blood pressure, other cardiovascular risk factors and state of arterial aging. You will also receive a detailed report with the results of the scans performed. In addition, the information obtained from this research project may contribute to medical progress and may help other patients in the future. You will not receive any economic benefit for the donation of the samples and the transfer of the data provided, nor will you have rights over possible commercial benefits of the discoveries that can be obtained as a result of the investigation carried out.

Data protection and confidentiality

All information about your results will be treated in a strictly confidential manner. Your data will be identified by means of a code, so that it does not include information that can identify you, and only the research team can relate that data to you. The research team assumes responsibility for the protection of personal data. Both your samples and your data will be kept under the appropriate security conditions and it is guaranteed that the subjects cannot be identified through means considered reasonable by people other than those authorized.

If the results of the study are subject to publication in scientific journals, personal data of the participants in this research will not be provided at any time. Your personal data will be protected in accordance with the provisions of Organic Law 3/2018, of December 5, on the Protection of Personal Data and guarantee of digital rights and Regulation (EU) 2016/679 of the European Parliament and of the Council of 27 April 2016 Data Protection (GDPR), having the right to access, rectify or cancel your data, you can limit the processing of data that is incorrect, request a copy or transfer the data that you have provided to a third party the study.

To exercise your rights, contact the research team whose data is specified at the end of this document. You also have the right to contact the Data Protection Agency if you are not satisfied.

Results Information

In the event that you request it, at the end of the study and in accordance with article 27 of Law 14/2007 on Biomedical Research, you can be provided with information on the results of this research work and on research studies in those who have used their samples.

In case of storage of samples

I consent to the storage and use of biological samples and associated data for future research under the conditions explained in this information sheet.

□ YES □ NO

In case of use of the data in future investigations

I consent to the future use of the data or samples that have been collected in the present research study to carry out other research related to the medical specialty or the research area of the present study.

□ YES □ NO

I agree that in the future my clinical history will be accessed again to collect data that are considered important for other research related to the medical specialty or research area of the present study.

□ YES □ NO

Contact details of the research team:

Name: Luis Garcia Ortiz.

Address: Primary Care Research Unit of Salamanca (APISAL)

CS. San Juan. Portugal Avenue 83, 2nd floor. 37005 Salamanca

Telephone: 923 291100-54750 / Email: apisal2020@gmail.com

INFORMED CONSENT

Intestinal microbiota and its relationship with lifestyles and arterial aging (MIVAS III Study)

Me (Name and Surname) ____

I have been able to ask questions about the study.

I have received enough information about the study.

I have read the information sheet given to me

I have spoken with the Investigator ___________________________________

I understand that my participation is voluntary.

I understand that I can withdraw from the study:

1st whenever you want

2nd Without having to explain

3rd Without having any negative impact

I voluntarily agree to participate in the Project and authorize the use of all information obtained. I understand that I will receive a signed copy of this informed consent.

_________________________ _________________________

Signature of participant Date

___________________________ _________________________

Name and signature of the researcher Date
